# One-step separation system of bio-functional lipid compounds from natural sources

**DOI:** 10.1016/j.mex.2021.101380

**Published:** 2021-05-08

**Authors:** Alexandros Tsoupras, Katherine M. Pappas, Theodore G. Sotiroudis, Constantinos A. Demopoulos

**Affiliations:** a Department of Biological Sciences, University of Limerick, Limerick, V94 T9PX, Ireland; b Health Research Institute, University of Limerick, Ireland; c Bernal Institute, University of Limerick, Ireland; d Department of Genetics and Biotechnology, Faculty of Biology, National and Kapodistrian University of Athens, 15701 Athens, Greece; e Institute of Chemical Biology, National Hellenic Research Foundation, Athens 116 35, Greece; f Faculty of Chemistry, National and Kapodistrian University of Athens, 15771 Athens, Greece

**Keywords:** HPLC, Reversed phase, Total lipids, Polar lipids, Glycolipids, Phospholipids, PAF, Phenolic compounds, Neutral lipids, Natural origin, PL(s), polar lipid(s), NL(s), neutral lipid(s), TL(s), total lipid(s), ω3 PUFA, omega-3 polyunsaturated fatty acids, PAF, platelet-activating factor, HPLC, high pressure (performance) liquid chromatography, LC, liquid chromatography, GC, gas chromatography, MS, mass spectra

## Abstract

Lipids are a very heterogeneous class of biomolecules with distinct structures and functions. Total lipids (TLs) obtained from natural sources are regularly further separated into lipid subclasses, with the two major ones being the polar lipids (PLs) and neutral lipids (NLs). Traditional analytical methods for fractionating TLs into NLs, PLs, and their subclasses, usually comprise difficult, costly and time-consuming steps.

Instead, several benefits and applications are derived by implementing a novel one-step semi-preparative and reversed-phase HPLC-analysis for separating TLs into all kinds of lipid subclasses. This method allows a one-step separation/fractionation of several subclasses of bio-functional PLs (i.e. phospholipids, glycolipids, phenolic compounds, N-acyl-homoserine-lactones, etc.) and NLs (i.e. triacylglycerols, fatty acids, esters, etc.) from TL-extracts of a natural source, prior to further testing them for their bio-functionality (i.e. in bioassays/cell models) and structure-activity relationships (i.e. LC-MS/GC-MS).

• This method can be applied in several natural sources, such as animal and marine sources, plants, microorganisms of biotechnological and agricultural interest, foods, beverages and related products, and by-products.
• This method can also be applied for separating specific bio-functional lipids from complex medical and pharmaceutical samples (i.e. cells, tissues, blood, plasma, liposomes, etc.), either for evaluating their role in diseases (i.e. PAF/PAF-like molecules) or by elucidating their protective roles (i.e. PLs rich in ω3 PUFA) for supplements and nutraceuticals’ applications.

## Graphical abstract

**Figure:**
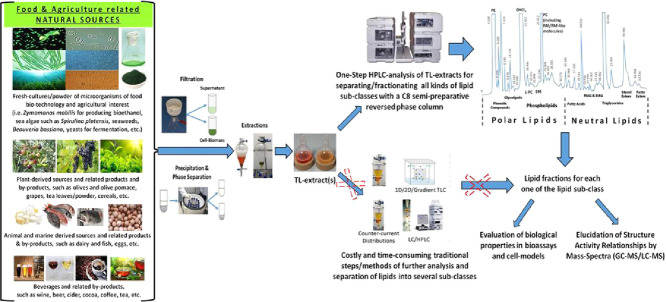

Specifications Table

**Table:** 

Subject Area:	• *Agricultural and Biological Sciences*
	• *Medicine and Dentistry*
	• *Pharmacology, Toxicology and Pharmaceutical Science*
More specific subject area:	*Separation & Analysis of Bioactive Lipids*
Method name:	*Applied one-step HPLC-separation of bio-functional lipids*
Co-Submission Paper	*Article title: Antithrombotic properties of Spirulina extracts against platelet-activating factor and thrombin* *Reference:****FBIO_100686*** *Journal title: Food Bioscience* *Article Number: 100686* *Status: Accepted for publication (in print)*
Name and reference of original method	*Tsoupras, A. B., Demopoulos, C. A. & Pappas, K. M. (2012). Platelet-activating factor detection, metabolism, and inhibitors in the ethanologenic bacterium Zymomonas mobilis, Eur J Lipid Sc Tech., 114(2), 123-133.*
Resource availability	*All reagents and instruments indicated are commercially available.*

## Method details

### Background

Lipids are a very heterogeneous class of biomolecules with a wide range of structures and functions. They can be divided into two major subclasses, namely the Neutral Lipid compounds (NLs) that are molecules with long hydrophobic hydrocarbon chains lacking a free polar group, and the Polar Lipid compounds (PL) that, apart from their hydrophobic hydrocarbon residues, they also bear at least one polar hydrophilic group. Classic examples of NLs are triacylglycerols, fatty acids and their esters, cholesterol and sterol esters, waxes, terpenes, etc. [1]. The most well known subclasses of PLs are the phospholipids that bear a phosphate head group with a hydrophilic residue within their structure (i.e. glycerophospholipids, such as phosphatidylcholine (PC), phosphatidylethanolamine (PE), phosphatidylserine (PS), phosphatidylglycerol (PG), phosphatidylinositol (PI), phosphatidic acid (PA), cardiolipin (CL), etc., and sphingophospholipids, such as compounds of the sphingomyelin (SM) family), the glycolipids that bear carbohydrate-group(s) (i.e.glycoglycerolipids and glycosphingolipids such as cerebrοsides, gangliosides, etc.) and phenolic compounds (i.e. flavones, flavanols, catechins, gallic acid, quercetin, resveratrol, etc.) [1].

Within these subclasses of lipids there are further differences in lipid structures and related bio-functionalities. For example, the biological importance of the majority of phopsholipids and glycolipids derives mostly from their amphiphilic properties [1]. The most characteristic example of this is that several of these lipids are essential components of cell membranes [1]. The lipid composition of biological membranes represents a taxonomic signature that distinguishes the different kingdoms of life. Differences between ester- and/or ether-bonded fatty acid chains at the glycerol/sphingosine backbone exist between different kinds of organisms, while the fatty acid composition of phopsholipids and glycolipids also varies depending on their origin [1]. Such PLs from microorganisms, plants or of animal origin, generally contain an unsaturated fatty acid in the *sn*-2 position, such as monounsaturated fatty acids (MUFA) (i.e. oleic acid) or polyunsaturated fatty acids (PUFA) (i.e. ω-6 PUFA such as linoleic acid and arachidonic acid, or ω3 PUFA such as α-linolenic acid (ALA), eicosapentaenoic acid (EPA) and docosahexaenoic acid (DHA)), whereas the *sn-*1 position predominantly carries a saturated fatty acid (SFA), such as stearic acid or palmitic acid [1]. The correct ratio of saturated to unsaturated fatty acids in the phospholipid membrane is essential to sustain membrane characteristics, since the fatty acid composition and degree of saturation directly affects the fluidity of the cell membrane [1]. Equally, the correct ratio can have a significant effect on cellular processes such as the formation of lipid rafts and other biological activities [1]. For example, it has been proposed that a low ratio of ω6/ω3 PUFA in diet has favorable effects against several chronic disorders, such as cardiovascular disease (CVD) and cancer [2], while PL-bearing ω3 PUFA such as ALA (usually from microorganisms and plants) or EPA and DHA (usually from marine sources) seem to possess distinct and favorable nutritional value and health benefits against these disorders in comparison to the fatty esters and triglycerides bearing such bioactive fatty acids [1], [3], [4], [5], [6], [7]

Another example of the crucial role played by the nature of bonds (ester or ether) and the fatty acid moieties with regard to PL bio-functionality, is offered by a specific subclass of PLs consisting of alcyl-acyl-phospholipids or alcyl-acyl-glycolipids [1], [3], [4], [5], [6], [7]. Such alkyl-PL usually bear hydrocarbon chains, saturated or unsaturated and with or without hydroxyl-groups, ether-linked to the *sn*-1 position of the glycerol/sphingo-backbone, instead of a fatty acid bound by ester bonds to the *sn*-1 position. Alkyl-PLs can be found as minor constituents of cell membranes in both prokaryotes and eukaryotes, but they are abundant in archaeal organisms. Some exist as bioactive molecules that seem to be maintained through evolution from archaeal to eukaryotic organisms because of their key roles in cell-cell signaling and related bioactivities, especially in eukaryotic organisms [1,^3^,4]. One such example is provided by plasmalogens and the platelet-activating factor, also known as PAF (1-O-alkyl-2-acetyl-sn-glyceryl-3-phosphorylcholine) [8], which is a potent inflammatory mediator involved in the immune response and in chronic inflammatory diseases [3,4], in addition to several other PLs with similar structure or activities to PAF that are called PAF-like molecules [3,[9], [10], [11], [12], [13].

It should be stressed that specific PLs belonging to the glycolipids and phopsholipids’ subclasses that are present in several natural sources (such as animal and marine sources, plants, microorganisms of biotechnological and agricultural interests, foods, beverages and related products and by-products), have been found to possess strong anti-platelet, anti-thrombotic and anti-inflammatory effects against inflammatory and thrombotic mediators like PAF and thrombin, but also against well established platelet agonists like adenosine diphosphate (ADP) and collagen [1,[3], [4], [5], [6], [7],[12], [13], [14], [15], [16], [17], [18], [19], [20], [21], [22], [23]. By such favorable bio-functionalities, these PLs have also exhibited promising outcomes against several inflammation-related chronic disorders, such as atherosclerosis and CVD, renal disorders, cancer, persistent infections (i.e. HIV-infection, periodontitis, leishmaniosis, etc.), where PAF is implicated [1,[3], [4], [5].

Bioactive PL molecules include but are not limited to the following: (i) phospholipids, such as phosphatidylcholines (PC), phosphatidylethanolamines, sphingomyelins (SM), and especially PAF-like molecules and/or those bearing ω3 PUFA in the *sn*-2 position of their structures, (ii) glycolipids, such as sulphoquinovosyldiacylglycerols (SQDG), mono/di-galactosyldiacylglycerols (MGDG/DGDG) and mono/di-glycodiacylglycerols, including glycolipids with palmitic acid at the *sn*-1 position and ALA/EPA/DHA esterified at the *sn*-2 position of their structures), (iii) cerebrosides and gangliosides, and (iv) other bioactive polar compounds such as phenolic compounds and phenol-lipids that usually migrate to the PL fraction of several separation procedures [1],[3], [4], [5], [6], [7],[12], [13], [14], [15], [16], [17], [18], [19], [20], [21], [22], [23].

Numerous phenolic compounds that are also present in several of the aforementioned natural sources, have also exhibited similar beneficial effects against inflammation and its related disorders, by favorably affecting biological pathways, including those of PAF [3,^4^,^16^,[17], [18], [19],[23], [24], [25]. Such phenolic compounds consist of flavonoids that are broadly classified into anthocyanidins (e.g., cyanidin, delphinidin, malvidin), flavanols (e.g., catechin, epicatechin), flavonols (e.g., quercetin, fisetin, kaempferol, and rutin), flavones (e.g., luteolin), but also of phenolic acids (i.e. caffeic, gallic, and quinic acids), and other similar compounds characterized by the presence of phenol units with polar substrates such as the OH-group [24], [25], [26]. These compounds, even though they are usually water soluble, they are more than often co-extracted with other PLs during TL-extract preparations and separations, because they exhibit slightly more to similar polarity with the more polar molecules of PLs.

Another interesting subclass of polar compounds that co-migrate with PLs at TL-extract separations, are the N-acyl-L-homoserine lactones (AHLs), which also have distinct bio-functionalities during cell-cell signaling and quorum sensing of microorganisms [27], [28], [29].

Taking into consideration all of the above, it is apparent that the majority of bioactive PL compounds are co-extracted into the TL- extract preparations derived from extractions of several of the aforementioned natural sources, independently of the extraction methods used. For instance, considerable amounts of various PL compounds have been found in TL extracts prepared by the use of highly non-polar solvents such as hexane implemented on fish oils [1]. Hexane-based extractions of marine-derived fish oils yield a vast majority of NL components, but also considerable amounts of bioactive PLs, which seem to be the ones providing most of the favorable health benefits of fish-oil consumption [1]. Similar outcomes are observed for the also highly heterogenic NL subclass of lipids, which, as aforementioned, contains triacylglycerols, fatty acids and their esters, cholesterol and sterol esters, waxes, terpenes, and other compounds of neutral polarity. Such NL content is often present in the TL content of complex natural sources such as the ones described [1].

Traditionally, TLs are obtained from a natural source, either by extraction (by using conventional methods of extraction such as the Bligh & Dyer method [6,^18^,30] or food grade ones [7]), or by applying other mechanical methods, such as specific pressure and temperature conditions during supercritical fluid extraction (SFE), subcritical water extraction (SWE), ultrasound-assisted extraction (UAE), and microwave-assisted extraction (MAE) [31]. Then, these TL preparations are usually further separated into the PL and NL subclasses by applying classic methods of lipid analysis, such as counter current distribution (CCD) by using conventional solvents [33] or food grade solvents and methodology [7], liquid column chromatography (LC), solid phase extractions (SPE), thin layer chromatography (TLC) in one or two dimensions (1D or 2D respectively), high pressure (performance) liquid chromatography (HPLC) of analytical grade, and several other similar methods [5], [6], [7],^10^,^11^,[15], [16], [17], [18], [19], [20], [21],[34], [35], [36], [37], [38].

Even when applying the aforementioned separation methods in any order, it is not guaranteed that a proper separation of specific lipid compounds from all lipid subclasses can be adequately achieved. Moreover, such a traditional approach usually demands high expertise and comprises several costly, time-consuming, and intricate steps that are not always successful. Particularly, when in the course of such separations several HPLC steps - of analysis are implemented, this usually requires the use of analytical grade columns and serial injections of low amounts of TL extracts from natural source samples in order to obtain good separations [5,^11^,^18^,[34], [35], [36], [37], [38]. Thus, to be able to analyze preparative amounts of such TL extracts, several repetitions are needed, which renders the scale laborious and expensive.

From all the above it is obvious that there is a need to reduce the degree of difficulty entailed in traditional methods applied for lipid separation, and this can be achieved by implementing approaches and methods of analysis consisting of as few as possible steps, without however compromising the efficacy of the separation. In the present study we evaluate the benefits and applications of a recently developed method for a one-step HPLC analysis and separation of TLs from natural sources. Our separation method resorts into several fractions of all major bioactive lipid sub-classes, which can be further corroborated by testing the fractions for their bio-functionality in specified bioassays and cell-models, and also by implementing structure – activity relationships through tandem or not mass spectra (LC-MS and/or GC-MS) analyses.

## Materials, methods and applications

### Materials and reagents

Standard phospholipids (PC, PE, L-PC, SM) and semi-synthetic cold and ^3^H-PAF (1-O-alkyl-2-acetyl-sn-glyceryl-3-phosphorylcholine), along with analytical reagents and organic solvents of HPLC- analytical purity, such as chloroform, methanol, water and acetonitrile, were purchased from Sigma Aldrich Co. (St. Louis, MO, USA).

### Method of analysis

#### Preparation of sample

TLs are obtained from a natural source, either by extraction (by using conventional methods of extraction such as the Bligh & Dyer method [6,^18^,30] or by utilizing food grade solvents and techniques [7]). In addition, other mechanical methods can also be used, such as SFE, SWE, UAE, and MAE [31].

Among all these methods, the Bligh & Dyer method [30] is the simplest extraction procedure for the preparation of the TLs extracts from solid or liquid natural sources [6,^18^,32]. More specifically, the TL extraction is achieved as previously described [18],[32]], by homogenisation of the sample in a monophasic system containing chloroform/methanol/water in a 1:2:0.8 (v/v/v) ratio. In the case of solid samples, tha sample is usually blendered within this solution and then filtration of the blendered extracts is usually conducted to separate the homogenate from the precipitated solid remnants of the sample, with filtering papers of 110 mm (Whatman, Maidstone, UK), under vacuum conditions by pumping in a Buchner-based filtering devices, as previously described [32]. The homogenate/filtrate from a sample (liquid or solid sample, respectively) is then transferred to a separatory funnel and addition of appropriate volumes of water and chloroform is then preformed in order to adjust the chloroform/methanol/water based homogenate at a ratio of 1/1/0.9 (v/v/v) to achieve phase separation with the TL being present in the lower phase. This phase is then gathered in round-bottom flasks and evaporated until dry on a flash rotary evaporator at 37°C under vacuum between 700-50 mbar (Buchi Rotavapor, Mason Technology Ltd., Dublin, Ireland), and then re-dissolved in a chloroform/methanol solution at a ratio of 1/1 (v/v) and transferred at a small glass tube, which is usually evaporated under ni-trogen stream. The obtained TL can then be weighted and stored under nitrogen at -20°C for max 8 weeks. Just before the HPLC analysis, the sample is dissolved again in a small volume of a chloroform/methanol solution at a ratio of 1/1 (v/v) at room temperature.

#### HPLC analysis

The one-step HPLC-separation procedure of TLs obtained from a natural source into several bio-functional lipid subclasses/fractions was performed at room temperature (20°C) by using a HP Series 1100 HPLC model (Hewlett Packard, Palo Alto, CA, USA), and the semi-preparative reversed phase column Luna 5u C8(2) 100A by Phenomenex (Torrance, CA, USA). A gradient distribution of HPLC-high-purity acetonitrile (ACN) and water (W) was used as a mobile phase, previously described by Tsoupras *et al.* [12]. However, any HPLC-instrumentation of same specifics and a similar separation regime may be applied for the purposes we describe.

**Fig. 1:**
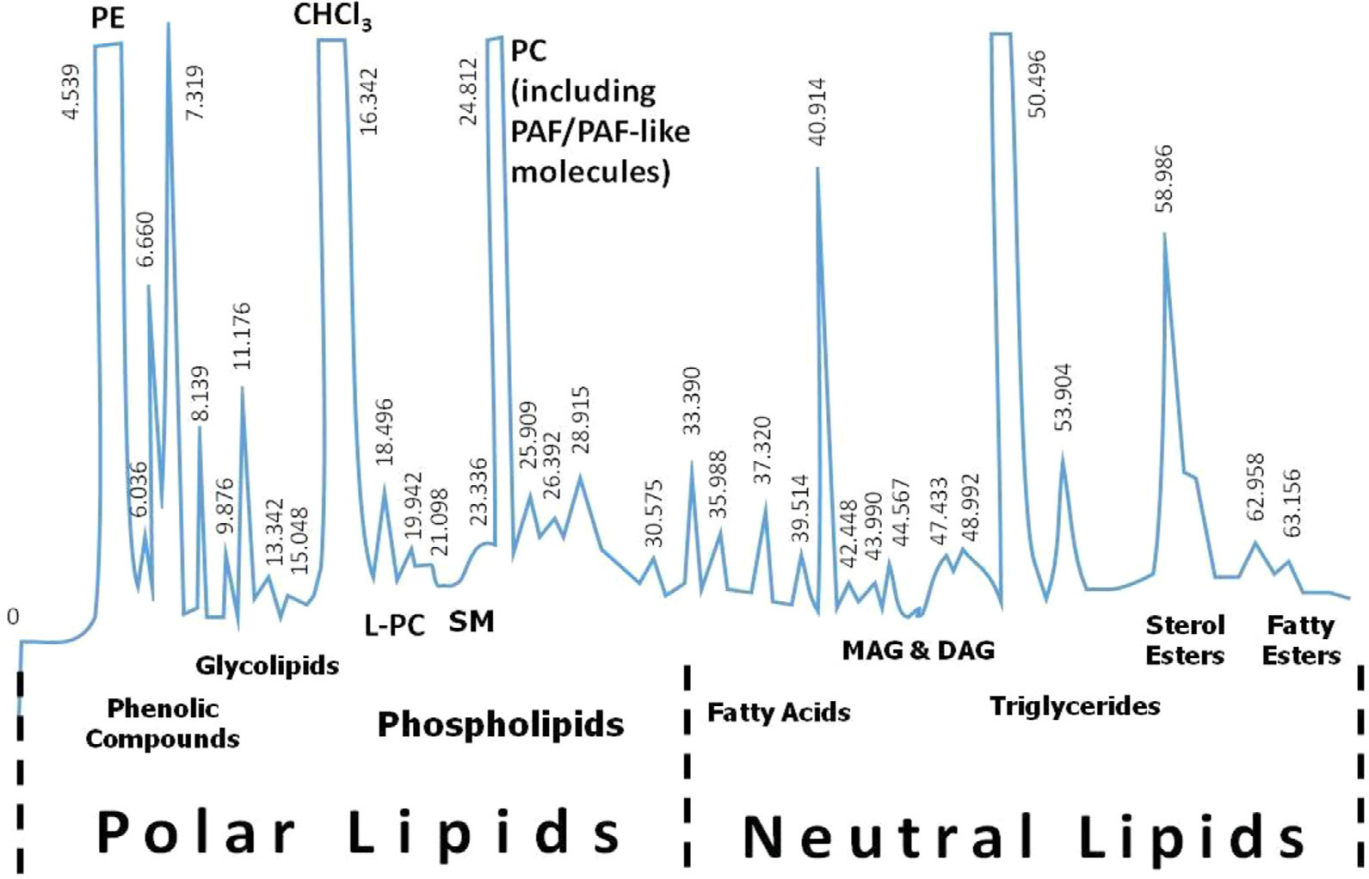
***Representative chromatograph of the One-Step HPLC-separation of total lipids from a natural source into the most important lipid subclasses.*** The HPLC purification procedure can be performed as previously described by Tsoupras et al. [12]. In the experimental conditions applied, several PL-subclasses are eluted first, such as phenolic compounds, glycolipids and phospholipids, while after 30-35 min of analysis NL-subclasses start to elute such as FFA, MAG, DAG, TAG, STE, esters of FA with fatty alcohols, waxes, etc. Which ones of these subclasses are present depends on the natural source from which the TLs and subsequently the PLs and NLs were obtained. HPLC: high pressure (performance) liquid chromatography; CAN: acetonitrile; W: water; PC: phosphatidylcholines; PE: phosphatidylethanolamines; L-PC: lyso-phosphatidylcholines; SM: sphingomyelins; PAF: platelet-activating factor; FA: fatty acids; FFA: free fatty acids; MAG: Monoalcylglycerol; DAG: dialcylglycerol; TAG: triglycerides; sterol esters (STE).

Briefly in our method, and after sample injection, a mobile phase of ACN:W at a 40:60 v/v ratio was used for 0-2 min, followed by a gradient for the next 2-26 min, which reached a final ACN:W 100:0 v/v ratio, kept until the end of the separation (80 min). The flow rate on all HPLC procedures was 3 mL/min. Prior to analysis, care was taken to dilute each sample in a solvent containing chloroform:methanol at a 1:1 v/v ratio and then inject for separation. In these conditions, and according to absorption values obtained by a UV-detector (208 nm), several PL subclasses were eluted first, while after 30-35 min of analysis the NL subclasses started to elute (Fig. 1).

In the case of PLs, phenolic-compounds are eluted in the first 5-10 min, such as flavonoids, catechins, resveratrol, quercetin, gallic acid, etc., followed by glycolipid compounds (10-15 min), such as digalactosyldiacylglycerol (DGDG), monogalactosyldiacylglycerol (MGDG),sulphoquinovosyldiacylglycerol (SQDG), cerebrosides (CER), gangliosides (GAN), etc. The presence of these subclasses depends on the natural source from which the TLs and subsequently the PLs were obtained. Then the solvent chloroform is eluted (15-17 min), and afterwards phospholipids (i.e. L-PC, SM, PC), until 30-35 min of retention time, with the exception of PE that is eluted early during separation (3-5 min). Semi-synthetic cold and ^3^H-PAF (1-O-alkyl-2-acetyl-sn-glyceryl-3-phosphorylcholine) and similar to PAF molecules, namely PAF-like molecules, are usually eluted in fractions with retention times similar to that of PC (depending on their fatty acid composition).

In the case of NLs and after 35 min, several subclasses are eluted, such as free fatty acids (FFA), monoacylglycerols (MAG), diacylglycerols (DAG), triglycerides (TAG), sterol esters (STE), esters of FA with fatty alcohols, and waxes, again, depending on the natural source of the TLs.

During our HPLC separation procedure, lipid fractions of lipid subclasses can manually be collected according to their absorption values under a UV-detector (208 nm), or collected automatically at specific time intervals (i.e. every 0.5 or 1 min), evaporated under a stream of nitrogen, re-dissolved in chloroform:methanol at a ratio of 1:1 v/v and stored in -20°C for further analysis.

### Advantages of the presented HPLC-methodology

After obtaining the TL-extract from a natural source, these TLs are usually further separated into the PL and NL subclasses by applying classic methods of lipid analysis, such as CCD, LC, SPE, TLC, HPLC of analytical grade, and other similar methods [5], [6], [7],^10^,^11^,[15], [16], [17], [18], [19], [20], [21],[30], [31], [33], [34], [35], [36], [37], [38], in any order. Such a traditional approach usually demands specific expertise and comprises of several costly, repetitive and time-consuming steps.Ιn several cases the guard pre-columns and even the column itself are saturated by bulky lipid samples that reduce the efficacy of the separation, and thus the use of analytical grade HPLC columns is further compromised.

Instead, the presented HPLC-separation method allows a one-step separation/fractionation of TLs extracted from natural sources, into several fractions of all major bioactive lipid sub-classes, such as bio-functional PLs (i.e. phospholipids, glycolipids, phenolic-compounds, N-acyl-homoserine-lactones, etc.) and NLs (i.e. triacylglycerols, fatty-acids, esters, etc.), without the need of previous separation techniques such as CCD, LC, SPE and TLC.

Furthermore, this HPLC-method has the advantage of using a semi-preparative column, and thus it can separate high amounts of dense TL-extracts into such bioactive lipid subclasses, by applying a low number of injections (1-2) in comparison to HPLC methods based in analytical grade columns where at least 10 injections are required for separating the same amount of TL-extract.

Finally, this one-step HPLC analysis and separation/fractionation of TL extracts has several applications if coupled with bioassays and use in cell-models, but also if combined in tandem with mass spectra analyses (LC-MS and/or GC-MS) for elucidating structure – activity relationships.

In addition, with this method the quantification of specific lipid molecules can also be achieved by the use of a calibration curve of either an external or an internal standard. For example, external PL standards from stock solutions and intermediate solutions can be prepared at 1 mg/mL and 20 μg/mL, respectively, in chloroform/methanol (1:1 v/v) and stored at −20°C under nitrogen. Serial dilutions of the intermediate solution in chloroform/methanol (1:1 v/v) are then made to give a six-point calibration curve with the use of the concentration ratio vs. peak area ratio for each PL class. In the case of quantifying bioactive lipid molecules with very low peak areas, such as the PAF molecule that is usually present in very low concentrations in relation to the other PLs in a TLs extract from a biological source (blood sample, tissue sample, cell samples) and thus provide very low peak areas, then alternatively an internal standard of semi-synthetic cold and ^3^H-PAF mixture can also be used by utilizing a liquid scintillation counter. In this case, the calibration curve is based on the concentration ratio vs. counts per minutes (cpm) ratio of the eluted molecule at the specific retention time for quantifying the concentration of bioactive lipid signaling molecules like PAF in a biological sample.

## Applications

### Separation of bioactive PL and NL subclasses from TLs of several natural sources

Οur proposed HPLC methodology has been applied efficiently for separating TL-extracts obtained from several microorganisms of agricultural, biotechnological and bio-pharmaceutical interest, into bioactive lipid-subclasses. By the implementation of the presented HPLC-method, the detection and separation of bioactive molecules from specific species of these microorganisms, was also achieved for the first time [12], [13], [14].

**Fig. 2:**
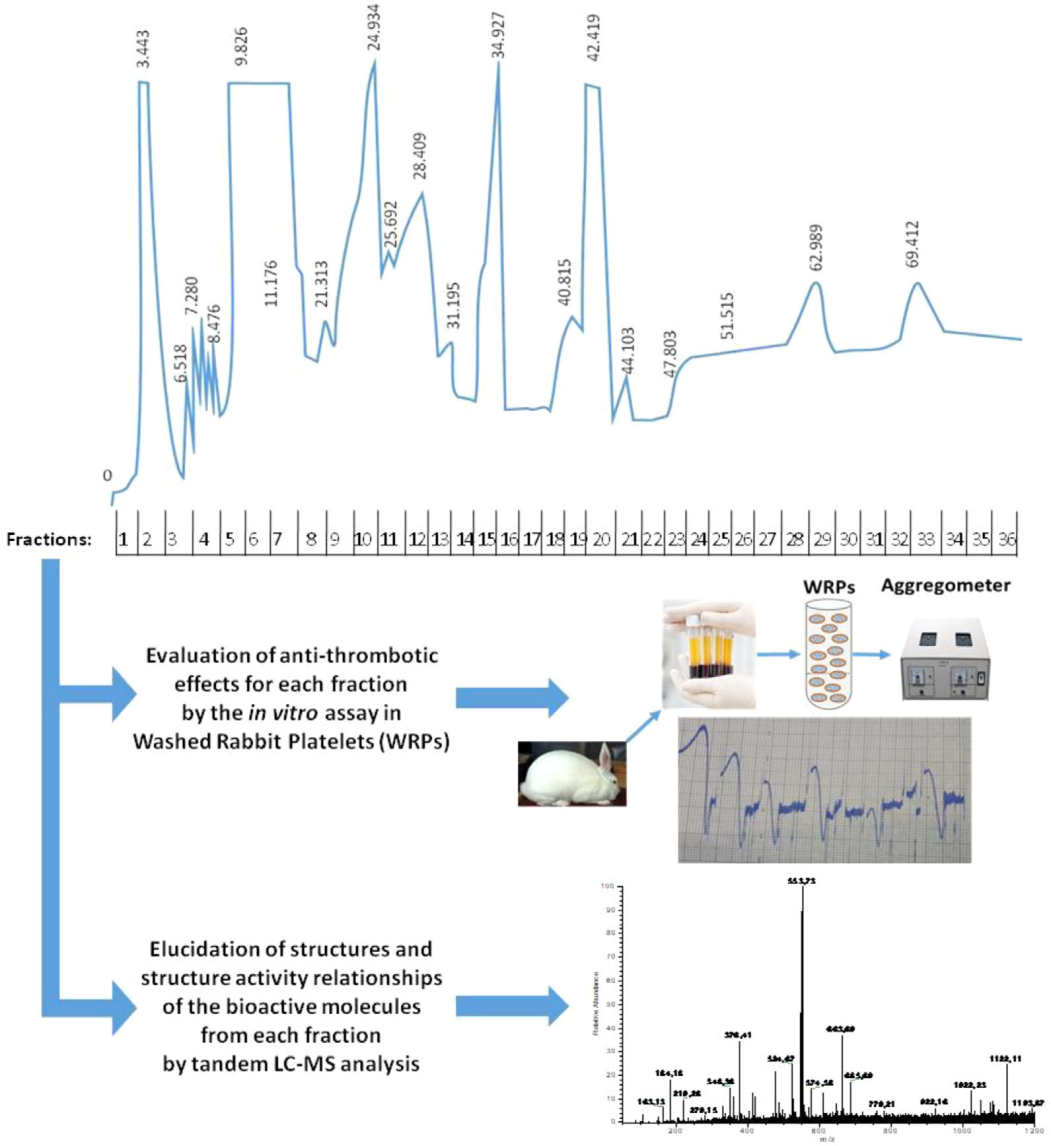
***Representative application of the One-Step HPLC-separation of total lipids from a natural source into fractions of bioactive lipid molecules with antithrombotic/thrombotic activities.*** The HPLC chromatogram depicts the separation of TL-extracts from the supernatant of *Spirulina platensis* cell cultures into fractions of bioactive lipid molecules with antithrombotic activities, as it was evaluated by tandem platelet aggregometry assays in washed rabbit platelets (WRPs) against platelet aggregation induced by the potent inflammatory and thrombotic mediators platelet-activating factor (PAF) and thrombin [13]. This experimental approach has also been used effectively in separating bioactive lipid molecules with antithrombotic/thrombotic properties from TL-extracts of other microorganisms too, such as *Zymomonas mobilis*[12] and *Beauveria Bassiana*[14], while if tandem with LC-MS an elucidation of the structures and structure-activity relationships can also be achieved [12].

For example, by applying the current HPLC-method for fractionating TL-extracts obtained from *Zymomonas mobilis*, an ethanologenic a-proteobacterium, into several lipid subclasses, coupled with aggregometry assays in platelets, it was found for the first time that this bacterium also contains bioactive PL subclasses with potent inhibitory effects against PAF (Fig. 2) [12]. Furthermore, by implementing LC-MS analytics to the *Z. mobilis* TL-extract fractions, it was also discovered that this bacterium synthesizes and secretes PAF, a signaling molecule that plays major role in eukaryotic cell-cell signaling (Fig. 2) [4,12]. These results revealed that apart from the well-established application of *Zymomonas* as a platform microorganism for large-scale bioethanol production, this interesting bacterium seems also to be a potential source of high-value bioactive compounds of interest to the food and healthcare industries, with obvious bio-pharmaceutical applications [12].

Another interesting example of the application of the presented HPLC-method was the fractionation of TL-extracts derived from sea-algae, such as *Spirulina*, into bioactive lipid subclasses [13]. Such marine microorganisms have been proposed as sustainable sources of several important nutrients. In addition to that, by applying the presented HPLC-method for separating TL-extracts obtained from *Spirulina platensis,* it was also found for the first time that this microalgae contain bioactive PL subclasses (e.g. phenolic compounds, SQDG and other glycolipids, PAF-like molecules of the PC and SM families, etc.), with potent inhibitory effects against both PAF and thrombin (Fig. 2) [13]. The results obtained by the application of this HPLC-method further support the use of such microalgae for the appropriate design and production of food supplements and nutraceuticals against inflammation-related chronic disorders associated with the PAF and thrombin pathways.

Finally, similar interesting results were obtained by applying this HPLC-method for fractionating TL-extracts from *Beauveria bassiana*, a widely used entomopathogenic fungus, into several subclasses of bioactive lipid compounds [14, under submission]. By coupling the HPLC lipid-fractionation method with aggregometry-related bioassays in platelets, the presence of several bioactive lipid molecules in *B. bassiana* culture supernatant with strong anti-inflammatory and anti-coagulant effects against PAF-activities and platelet-aggregation was observed, which provide new perspectives and putative future applications for this agriculture-related fungus.

## Future applications

**Fig. 3:**
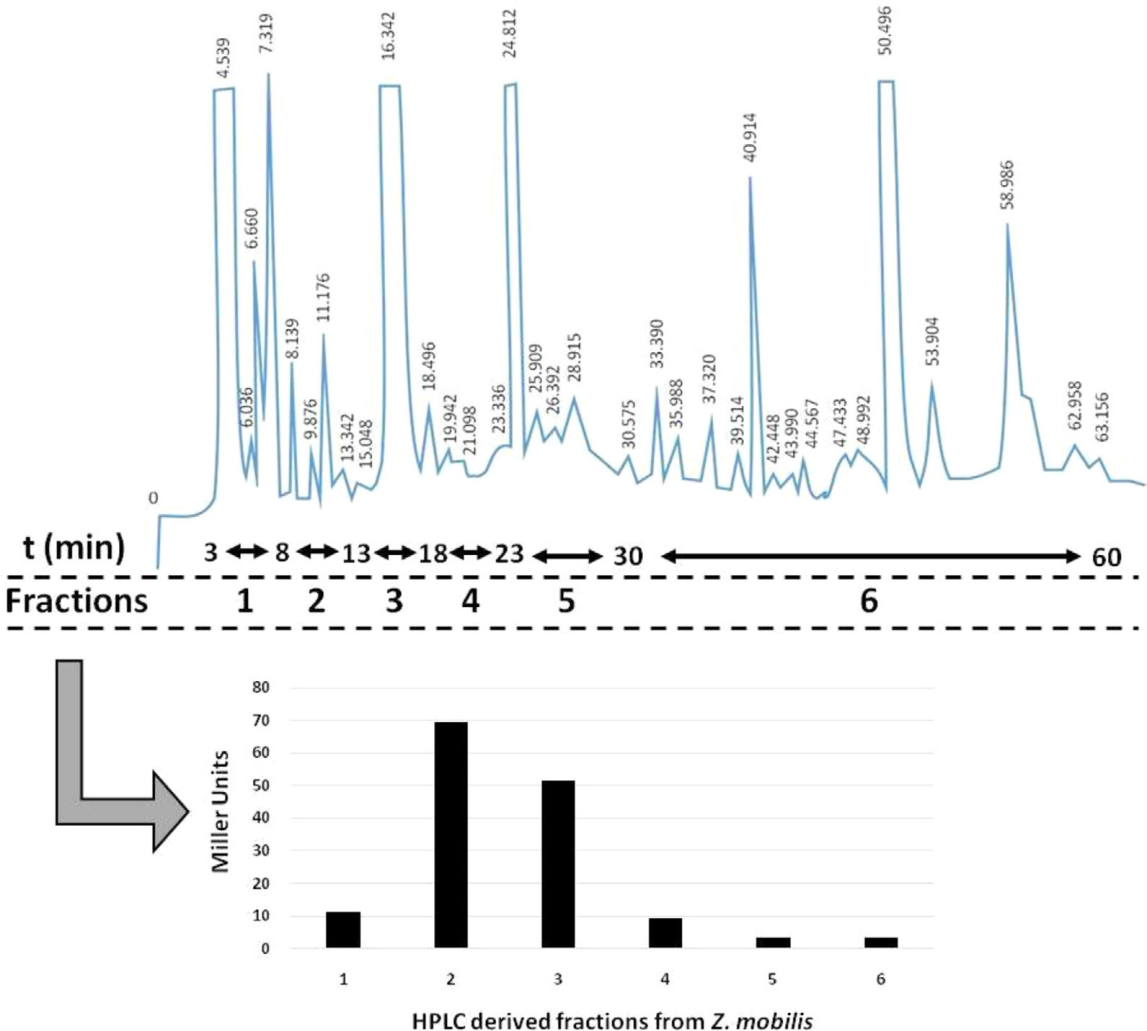
***Representative application of the οne-step HPLC separation of TLs from Zymomonas mobilis into molecular species exhibiting proteobacterial pheromone (AHL) bioactivity.*** The HPLC chromatogram depicts *Z. mobilis* culture-supernatant extract separation into fractions exhibiting AHL activity. The latter is evaluated by a tandem bioassay procedure making use of an indicator *Agrobacterium tumefaciens* strain and β-galactosidase (Miller-unit) measurements [39]. In this experimental procedure, fractions 2 and 3, in which standard 3-oxo-C6-, 3-oxo-C8- and 3-oxo-C10-AHLs are usually eluted (data in submission), exhibited the strongest bioactivity, suggesting that compounds of AHL nature may be produced and secreted from *Z. mobilis*.
• The presented HPLC-separation method can also be applied effectively in a one-step way of separating bioactive lipid compounds from dense TL extracts of several other natural sources, such as animal and marine sources, plants, several other microorganisms of agricultural, biotechnological and biopharmaceutical interests, and several foods, beverages and related products and by-products (Fig. 2).
• The presented HPLC method for a one-step lipid fractionation from TL extracts, can also accomplish a more effective separation of bioactive lipids and lipid inflammatory mediators (i.e. PAF and PAF-like molecules) from dense and complex medical and pharmaceutical samples (i.e. cells, tissues, blood, plasma, liposomes, etc.) for evaluating and monitoring their role in inflammation-related disorders in which PAF is associated [3,^4^,^10^,^11^,39] or for elucidating and monitoring the protective roles of supplemented or nutrition-derived bio-functional lipids (i.e. PL rich in ω3 PUFA, vitamin D, etc.) [3], [4], [5],[22], [23],40] related to nutraceutical and bio-pharmaceutical applications (Fig. 2).

• Another interesting application of this one-step HPLC-separation method is the separation of specific bioactive subclasses of lipid-molecules implicated in bacterial quorum sensing [28]. Preliminary assays in *Z. mobilis* supernatant extracts, revealed the presence of fractions exhibiting N-acyl-homoserine lactone (AHL) activity or even fractions exhibiting inhibition of such an activity (Fig. 3, data in submission), as shown by coupling the fractionation with an AHL-indicative *in vivo* bioassay [39]. Such an analysis promotes the realization that industrial biotechnological platforms, such as *Z. mobilis*, may engage in coordinated community behaviors, a fact highly important when monoculture or mixed-culture fermentation regimes are taken into account.

## Declaration of Competing Interest

The authors declare no conflict of interest.
